# Cytogeography of Callisia section Cuthbertia (Commelinaceae)

**DOI:** 10.3897/CompCytogen.v11i4.11984

**Published:** 2017-09-01

**Authors:** Iwan E. Molgo, Douglas E. Soltis, Pamela S. Soltis

**Affiliations:** 1 Florida Museum of Natural History, University of Florida, Gainesville, Florida 32611-7800 U.S.A.; 2 Department of Biology, University of Florida, Gainesville, Florida 32611 U.S.A.; 3 Genetics Institute, University of Florida, Gainesville, Florida 32608 U.S.A.; 4 Biodiversity Institute, University of Florida, Gainesville, Florida 32611 U.S.A.

**Keywords:** chromosome counts, cytotypes, endemic, Florida scrub vegetation, flow cytometry, genome size, polyploidy, sandhill vegetation, Southeastern United States

## Abstract

Determining the distribution of cytotypes across the geographic distribution of polyploid complexes can provide valuable information about the evolution of biodiversity. Here, the phytogeography of cytotypes in section Cuthbertia (Small, 1903) Hunt, 1986 is investigated. A total of 436 voucher specimens was georeferenced; 133 new specimens were collected. Based on flow cytometry data, DNA content of all cytotypes in section Cuthbertia was estimated. Utilizing chromosome counts and flow cytometric analysis, cytotype distribution maps were generated. Two disjunct groups of populations of diploid *Callisia graminea* (Small, 1903) Tucker, 1989 were discovered; tetraploid *C. graminea* ranges broadly from the coastal plain of North Carolina through central Florida. One hexaploid *C. graminea* individual was recorded in South Carolina, and numerous individuals of hexaploid *C. graminea* were found in central Florida. Diploid *C. ornata* (Small, 1933) Tucker, 1989 occurs in eastern Florida; previously unknown tetraploid and hexaploid populations of *C. ornata* were discovered in western and central Florida, respectively. Diploid *C. rosea* (Ventenat, 1800) Hunt, 1986 occurs in Georgia and the Carolinas, with populations occurring on both sides of the Fall Line. The cytotype and species distributions in *Callisia* are complex, and these results provide hypotheses, to be tested with morphological and molecular data, about the origins of the polyploid cytotypes.

## Introduction

Polyploidy (whole-genome duplication) is a speciation mechanism that is a major evolutionary force; in fact, all angiosperms have undergone at least one ancient polyploidy event (Jiao et al. 2011, *Amborella* Genome Project 2013), and polyploidy has been a key driver of angiosperm diversity (De Bodt et al. 2005, Soltis et al. 2009, Soltis and Soltis 2009, Soltis and Soltis 2016, Tank et al. 2015).

Polyploids are classified in two major categories: allopolyploids and autopolyploids. Allopolyploids are by far the more studied form and arise via hybridization between species, whereas autopolyploids originate from the multiplication of genomes within a single species. An autopolyploid is frequently considered as a cytotype within a species along with its diploid progenitor, as in *Galax urceolata* (Poiret, 1804) Brummitt, 1972 (Baldwin 1941, Stebbins 1950), *Chamerion angustifolium* (Linnaeus, 1753) Holub, 1972 (Mosquin 1967), *Heuchera grossulariifolia* Rydberg, 1900 (Wolf et al. 1990), and *Vaccinium corymbosum* Linnaeus, 1753 (Camp 1945, Krebs and Hancock 1989). However, autotetraploids are occasionally recognized as species distinct from their diploid parent, such as *Zea perennis* (Hitchcock, 1922) Reeves & Mangelsdorf, 1942 (Iltis et al. 1979, Tiffin and Gaut 2001) and *Tolmiea menziesii* Torrey & Gray, 1840 (Judd et al. 2007). Lumping diploid progenitors with their multiple derivative cytotypes into a single species may mask evolutionary lineages and grossly underestimate biodiversity (Soltis et al. 2007).

To gain a better assessment of biodiversity and to guide conservation efforts for species of interest, data on both evolutionary and life-history characteristics are needed. Callisia section Cuthbertia (Commelinaceae) from the southeastern U.S.A. comprises a polyploid complex, with species of conservation concern, but the extent of polyploidy and the geographic distribution of cytotype diversity are unknown.

*Callisia* Loefling,1758 is one of 39 genera in subfamily Commelinoideae (Burns et al. 2011) and is placed in tribe Tradescantieae subtribe Tradescantiinae. *Callisia* comprises approximately 23 species in six sections (*Hadrodemas* (Moore, 1963) Hunt, 1986, *Cuthbertia* (Small, 1903) Hunt, 1986, *Lauia* Hunt, 1986, *Brachyphylla* Hunt, 1986, *Leptocallisia* Bentham & Hooker, 1883, and *Callisia*) (Hunt 1986, Tucker 1989). Of these sections, *Cuthbertia* is endemic to the U.S.A., and *Brachyphylla*, *Leptocallisia*, and *Callisia* also have members that occur in the U.S.A. (Tucker 1989). The remaining two sections (*Lauia* and *Hadrodemas*) occur in Central America, South America, and the Caribbean. In recent phylogenetic analyses, *Callisia* is not monophyletic (Bergamo 2003, Burns et al. 2011), although, significantly, section Cuthbertia is monophyletic in all analyses (Bergamo 2003, Burns et al. 2011, Hertweck and Pires 2014).

Callisia section Cuthbertia consists of three morphologically distinct species (*C. graminea*, *C. ornata*, and *C. rosea*) that are endemic to the southeastern U.S.A. and have a base chromosome number of *x* = 6 (Giles 1942, 1943). *Callisia graminea* (Small, 1903) Tucker, 1989, the grassleaf roseling, occurs from the southern border of Virginia through central Florida. Giles (1942, 1943) reported three ploidal levels (2*x*, 4*x*, and 6*x*) for this species and encountered a single triploid individual in Hoke County, NC. Based on cytological criteria, the tetraploid was interpreted as an autopolyploid derivative of diploid *C. graminea* (Giles 1942, 1943). The nature of polyploidy in hexaploid *C. graminea* is not clear. Within *C. graminea*, two forms have been described: C. graminea forma graminea has pink flowers with anthocyanin pigments, and C. graminea forma leucantha (Lakela, 1972) Tucker, 1989 has white flowers and was described from two diploid cuttings (Lakela 1972). *Callisia ornata* (Small, 1903) Tucker, 1989 (Florida scrub roseling), a diploid (Giles unpublished), is endemic to central to southern Florida. *Callisia rosea* (Ventenat, 1800) Hunt, 1986 (Piedmont roseling) is a diploid (Anderson and Sax 1936), with a distribution from North Carolina to Georgia.

Although earlier studies (e.g., Giles 1942, 1943) provided the general pattern of species distributions and cytotypic diversity, the extent of cytotypic variation within and among species has not been examined in detail. Additional sampling of both populations and species is required to understand the extent and distribution of cytological variation in this clade. In this study, numerous new field collections were made, and known populations of Callisia section Cuthbertia were revisited; with the use of both traditional chromosome counts and flow cytometry, the ploidy of samples spanning the entire range of Callisia section Cuthbertia was investigated. Distribution maps of cytotypes and species were generated based on the cytological data obtained here, enabling future studies of phylogeny and polyploid origins in Callisia section Cuthbertia.

## Materials and methods

### Georeferencing

To obtain locality data for *Callisia graminea*, *C. ornata*, and *C. rosea*, voucher specimens were examined from the following herbaria: GA, USCH, NCU, DUKE, US, AAH, FLAS, FSU, VSC, and SFU (codes follow Thiers 2016). The locality of each specimen was georeferenced by manually incorporating the label data into the web applications ACME mapper 2.1 (Poskanzer 2001) and/or GEOLocate (Rios and Bart 2010). Additional localities were obtained from the Master’s Thesis of A. Kelly (1991) and personal communications with members of the Florida Native Plant Society and photographers from Flickr.com. In all, 436 specimens were georeferenced from herbarium specimens and observation records. (See supplementary file 1: Table 1 for georeferenced data points.) The data points were used to produce a distribution map using ArcGIS 10.4 (ESRI 2016) and to locate known populations and contact zones of all three species and their cytotypes.

**Table 1.** Populations used in this study. Geographic location, ploidy, number of plants of each ploidy, total number of analyzed individuals, and voucher information for 133 populations of *Callisia graminea* (G), *C. ornata* (O), and *C. rosea* (R) from the southeastern United States. * indicates a new locality with voucher specimen.

				Geographic coordinates		Ploidy / Number of plants				
Population	Locality	State	County	Latitude / Longitude	2*x*	4*x*	6*x*	*N*	Voucher no.
*Callisia graminea* (Small) G. Tucker										
G-1*	Gainesville Regional Airport	FL	Alachua	29°42.01'N, 082°15.72'W		1		3	307
G-2	Jct. Tower Rd. and SW 8 Ave	FL	Alachua	29°38.63'N, 082°25.24'W		1		4	223
G-3	Morningside Nature Center	FL	Alachua	29°39.56'N, 082°16.45'W		1		1	234
G-4	Jct. Hwy 200 and CR. 491	FL	Citrus	28°58.51'N, 082°21.84'W		1		2	229
G-5*	Along Rod Rd.	FL	Clay	30°01.52'N, 081°51.95'W		1		1	225
G-6	Golden Branch Head State Park	FL	Clay	29°50.75'N, 081°57.04'W		1		2	309
G-7	Silver Sand Lake Rd.	FL	Clay	29°47.49'N, 081°58.32'W		1		4	311
G-8*	Tate Hell State Forest along New River	FL	Franklin	29°52.42'N, 084°41.79'W		1		4	306
G-9*	Richloam State Forest/Dark Stretch Rd.	FL	Hernando	28°29.10'N, 082°08.87'W			1	6	349
G-10*	Edwards Rd., Lady Lake	FL	Lake	28°54.12'N, 081°53.40'W			1	3	235
G-11*	Lake Griffin State Park	FL	Lake	28°52.31'N, 081°53.41'W			1	3	236
G-12*	Seminole State Forest along Co. Rd. 42	FL	Lake	29°00.82'N, 081°31.05'W		1	1	3	345
G-13*	Seminole State Forest	FL	Lake	28°49.31'N, 081°28.01'W		1		1	362
G-14*	Lake Norris Rd.	FL	Lake	28°54.89'N, 081°32.41'W		1		1	363
G-15*	ATV trail at Ocala National Forest	FL	Marion	29°21.76'N, 081°44.21'W		1		1	230
G-16	Silver River State Park	FL	Marion	29°12.15'N, 082°02.77'W		1		4	348
G-17*	Along Mason Rd.	FL	Putnam	29°42.50'N, 082°00.77'W		1		2	224
G-18*	Ordway Biological Center H1 & H2 area	FL	Putnam	29°41.70'N, 081°57.87'W		1		2	302
G-19*	Etoniah Creek State Forest	FL	Putnam	29°46.43'N, 081°51.91'W		1		3	308
G-20	Dunns Creek State Park entrance Sisco Rd.	FL	Putnam	29°31.84'N, 081°35.34'W		1		4	310
G-21*	Welaka State Forest	FL	Putnam	29°28.24'N, 081°39.37'W		1		2	360a
G-22	Along State Rd. 46	GA	Bulloch	32°20.94'N, 081°50.57'W		1		3	242
G-23	Jct. Hwy 185 and Turkey Ridge Dr.	GA	Charlton	30°24.76'N, 082°11.70'W		1		2	317
G-24*	General Coffee State Park	GA	Coffee	31°31.50'N, 082°46.33'W		1		1	318
G-25	N. Connector Rd./206 Jct. 135	GA	Coffee	31°32.27'N, 082°46.33'W		1		3	319
G-26*	George Smith State Park	GA	Emanuel	32°32.64'N, 082°07.32'W		1		6	241
G-27*	Ochicoo Preserve, Halls Bridge Rd.	GA	Emanuel	32°31.73'N, 082°27.38'W		1		4	320
G-28	Fort Stewart	GA	Evans	32°06.92'N, 081°47.10'W		1		4	243
G-29*	Conway CT./Interstate Parkway	GA	Richmond	33°29.24'N, 082°06.12'W		1		1	322
G-30	Fort Gordon	GA	Richmond	33°23.33'N, 082°14.56'W					239
G-31*	Singletary Lake State Park	NC	Bladen	34°35.41'N, 078°26.87'W		1		3	263
G-32*	Jones Lake State Park	NC	Bladen	34°42.11'N, 078°37.22'W		1		3	268
G-33*	Jones Lake State Park	NC	Bladen	34°42.11'N, 078°37.22'W		1			269
G-34*	Along NC 242 near Jones Lake State Park	NC	Bladen	34°42.00'N, 078°36.35'W		1		2	270
G-35*	Along NC 242 N. of Jones Lake State Park	NC	Bladen	34°45.40'N, 078°36.56'W		1		5	271
G-36*	White Lake, along NC 741, Barnes Food Co.	NC	Bladen	34°39.41'N, 078°30.17'W		1		5	272
G-37*	Jones Lake State Park. campsite	NC	Bladen	34°40.79'N, 078°35.99'W					274
G-38*	Along Burney Rd. underneath powerline	NC	Bladen	34°44.38'N, 078°43.68'W		1		4	334
G-39*	River Rd., underneath powerline	NC	Bladen	34°46.18'N, 078°47.24'W		1		3	335
G-40	Bay Tree Lake State Park/undeveloped	NC	Bladen	34°40.22'N, 078°25.66'W		1		6	261
G-41	Along Hwy 41 close to Bay Tree Lake State Park	NC	Bladen	34°41.21'N, 078°25.26'W		1		3	262
G-42	Along Hwy 11 towards Delco under powerline	NC	Bladen	34°24.61'N, 078°15.60'W		1		4	266
G-43	Along Jessup Pond	NC	Bladen	34°51.72'N, 078°43.76'W					275
G-44	Lake Waccamaw State Park.	NC	Columbus	34°16.73'N, 078°27.89'W					267
G-45*	Mack Simmons Rd.	NC	Cumberland	34°54.45'N, 078°44.20'W					276
G-46*	Along NC 210, Jct. with Sidney Bullard Rd.	NC	Cumberland	34°58.69'N, 078°43.84'W		1		4	278
G-47*	Ft. Bragg/John Mill Rd.	NC	Cumberland	35°10.70'N, 079°05.39'W	1			3	341
G-48*	Ft. Bragg/NE. training/Mc Closkey Rd.	NC	Cumberland	35°09.84'N, 078°56.97'W	1			3	342
G-49	Cedar Creek Rd., Tatum farm	NC	Cumberland	34°56.32'N, 078°44.58'W		1		1	277
G-50	Along Dunns Rd./NC 301	NC	Cumberland	35°06.42'N, 078°46.52'W					279
G-51	Open Area along NC 24	NC	Harnett	35°15.61'N, 079°02.47'W	1			3	284
G-52	Along Rockfish Rd.	NC	Hoke	34°59.32'N, 079°05.82'W	1			3	286
G-53	In open area along Red Springs Rd.	NC	Hoke	34°52.38'N, 079°12.17'W	1			4	287
G-54*	Weymouth Sandhill Nature Preserve	NC	Moore	35°08.95'N, 079°22.10'W	1			3	288
G-55	Along Riverview Dr.	NC	Moore	35°11.48'N, 079°10.94'W	1			3	285
G-56	Along NC 11/ Hwy 53	NC	Pender	34°29.72'N, 078°11.49'W		1		3	264
G-57	Along NC 11/ Hwy 53	NC	Pender	34°29.72'N, 078°11.49'W		1		1	265
G-58*	Grey Woods Rd.	NC	Richmond	34°57.52'N, 079°38.47'W	1			3	297
G-59*	Sandhills Game Land	NC	Richmond	35°01.83'N, 079°36.70'W	1			2	336
G-60*	Sandhills Game Land/442/Ledbetter Rd.	NC	Richmond	35°03.62'N, 079°38.09'W	1			3	337
G-61*	Sandhills Game Land	NC	Richmond	34°58.61'N, 079°30.42'W	1			2	338
G-62*	Sandhills Game Land SR 1331, 15/501	NC	Richmond	34°58.50'N, 079°26.93'W	1			2	339
G-63*	Sandhills Game Land, Aberdeen Rd./Hill Creek Rd.	NC	Richmond	34°59.49'N, 079°26.76'W	1			3	340
G-64	Sandhills Game Land along McDonald Church Rd.	NC	Richmond	35°01.24'N, 079°37.18'W	1			2	290
G-65	NC Hwy 177	NC	Richmond	34°50.41'N, 079°45.54'W		1		1	295
G-66	Along Saint Stevens Church Rd.	NC	Richmond	34°49.82'N, 079°50.55'W	1			1	296
G-67	NC 242, 0.3 mi N. of Cumberland Co. line	NC	Sampson	34°53.35'N, 078°31.28'W		1		3	273
G-68	Along Spiveys Corner Hwy.	NC	Sampson	35°10.72'N, 078°28.65'W	1			2	280
G-69	Edge camp Mackall along Aberdeen Rd.	NC	Scotland	35°00.84'N, 079°26.70'W	1			2	289
G-70	Along 1328, Hoffman Rd./Butler Rd.	NC	Scotland	34°59.14'N, 079°31.99'W	1			2	291
G-71	Along Peach Orchard Rd. under powerline	NC	Scotland	34°55.77'N, 079°23.86'W	1			3	292
G-72	Along US 401 and forest edge	NC	Scotland	34°50.49'N, 079°23.98'W	1			1	293
G-73	Along forest edge of Hamlet Rd.	NC	Scotland	34°48.01'N, 079°38.03'W	1			2	294
G-74	Along Piney Grove Church Rd.	NC	Wayne	35°17.32'N, 077°50.92'W	1			1	281
G-75*	Aiken State Park	SC	Aiken	33°32.55'N, 081°28.92'W		1		4	324
G-76*	Parcel at Jct. Hwy 283 & US 1/Columbia Hwy N	SC	Aiken	33°36.11'N, 081°41.04'W		1		5	325
G-77	Aiken Gopher Tortoise Heritage Preserve	SC	Aiken	33°30.00'N, 081°24.52'W		1		1	231
G-78*	Carolina Sandhills National Wildlife Refuge	SC	Chesterfield	34°31.46'N, 080°13.63'W	1			3	331
G-79*	Sandhill State Forest	SC	Chesterfield	34°33.37'N, 080°03.84'W	1			3	332
G-80*	H. Cooperblack Jr. Memorial trail/James Rd.	SC	Chesterfield	34°34.03'N, 079°55.75'W	1			2	333
G-81	Along Hwy 102	SC	Chesterfield	34°38.30'N, 080°05.22'W	1			5	249
G-82	Teals mill Rd./Cheraw State Park	SC	Chesterfield	34°37.25'N, 079°56.70'W	1	1		3	250
G-83	W. Old Camden Rd.	SC	Chesterfield	34°22.28'N, 080°16.92'W		1		3	252
G-84	US 1	SC	Chesterfield	34°26.17'N, 080°17.44'W		1		2	253
G-85	Along Old Stagecoach Rd.	SC	Chesterfield	34°20.96'N, 080°21.27'W		1		3	254
G-86	Along Old Georgetown Rd. E.	SC	Chesterfield	34°22.99'N, 080°23.29'W		1		1	255
G-87	Co. Rd. S. 18-137	SC	Dorchester	32°54.02'N, 080°23.11'W		1		4	248
G-88	Tillman Sand Ridge Heritage Preserve, Sandhill Rd.	SC	Jasper	32°29.69'N, 081°11.55'W		1		5	247
G-89*	Along Jefferson Davis Hwy/US 1	SC	Kershaw	34°18.73'N, 080°32.49'W					256
G-90*	Goodale State Park	SC	Kershaw	34°17.42'N, 080°31.55'W		1		3	329
G-91*	Jefferson Davis Hwy/US 1	SC	Kershaw	34°22.04'N, 080°25.92'W		1		4	330
G-92*	Lee State Park	SC	Lee	34°11.81'N, 080°11.36'W		1		3	251
G-93	Shealy’s Pond Heritage Preserve	SC	Lexington	33°51.82'N, 081°14.19'W		1		1	232
G-94	Peachtree Rock Preserve	SC	Lexington	33°49.71'N, 081°12.11'W		1		1	233
G-95*	Ft. Jackson, Area 26 B firebreak 16	SC	Richland	34°00.85'N, 080°47.40'W		1		2	257
G-96*	Ft. Jackson, Area 34 B near Chauers Pond Rd.	SC	Richland	34°02.36'N, 080°43.30'W		1		3	258
G-97*	Ft. Jackson, Area 11 E. of Wildcat Rd.	SC	Richland	34°05.06'N, 080°50.61'W		1		2	259
G-98	Ft. Jackson, S. edge of pond of Westons Recreation	SC	Richland	33°59.96'N, 080°50.03'W		1		1	260
G-99	Sesquicentennial State Park	SC	Richland	34°05.82'N, 080°54.57'W		1		3	326
G-100*	Sesquicentennial State Park	SC	Richland	34°04.92'N, 080°54.38'W		1	1	4	327
G-101	Faunas Rd.	SC	Richland	34°08.34'N, 081°02.33'W		1		5	328
G-102*	Forks of River Rd.	VA	Southampton	36°33.85'N, 076°55.96'W	1			2	282
G-103	Suffolk City, DCR	VA	Suffolk City	36°33.77'N, 076°54.82'W					283
*Callisia ornata* (Small) G. Tucker										
O-1*	Turkey Creek Sanctuary	FL	Brevard	28°01.01'N, 080°36.18'W	1			1	315
O-2*	Sebastian State Park	FL	Brevard	27°50.19'N, 080°31.56'W	1			2	361
O-3	Wickham Park	FL	Brevard	28°09.64'N, 080°39.54'W	1			1	314
O-4*	Highlands State Park	FL	Highlands	27°28.85'N, 081°31.57'W		1		4	301
O-5	Sebring Amtrak Station	FL	Highlands	27°29.75'N, 081°26.06'W					298
O-6	Lake June in Winter Scrub State Park	FL	Highlands	27°17.83'N, 081°25.14'W		1		2	300
O-7	Little Manatee State Park/Mustang trail	FL	Hillsborough	27°40.08'N, 082°22.1'W		1		4	350
O-8	Little Manatee State Park/Dude trail	FL	Hillsborough	27°39.93'N, 082°22.38'W		1		3	351
O-9*	Seminole State Forest/entrance Brantley Branch Rd.	FL	Lake	28°53.20'N, 081°27.60'W			1	4	343
O-10*	Seminole State Forest/the Simson track	FL	Lake	28°52.94'N, 081°31.08'W			1	4	344
O-11*	Seminole State Forest/Warea tract	FL	Lake	28°29.99'N, 081°40.03'W			1	3	346
O-12*	Lake Louisa State Park/Primitive campsite	FL	Lake	28°27.17'N, 081°44.13'W			1	4	347
O-13	Jonathan Dickinson State Park/Nature trail picnic area	FL	Martin	26°59.58'N, 080°08.83'W	1			4	353
O-14*	Tiger Creek Preserve along Pfundstein Rd.	FL	Polk	27°48.41'N, 081°29.81'W		1		1	228
O-15*	Arbuckle State Forest, School Bus Rd.	FL	Polk	27°39.75'N, 081°23.84'W	1			3	316
O-16*	Lake Kissimmee State Park, Buster Island	FL	Polk	27°55.39'N, 081°21.82'W	1			2	354
O-17*	Lake Kissimmee State Park, Catfish Creek	FL	Polk	27°57.84'N, 081°22.77'W	1			5	355
O-18*	Lake Kissimmee State Park Main entrance	FL	Polk	27°57.91'N, 081°28.34'W	1			5	356
O-19*	Welaka State Forest	FL	Putnam	29°28.24'N, 081°39.37'W	1			1	360B
O-20	Dunns Creek State Park entrance Sisco Rd.	FL	Putnam	29°33.34'N, 081°34.94'W	1			2	312
O-21	Oscar Scherer State Park along Legacy trail	FL	Sarasota	27°10.17'N, 082°27.41'W		1		5	352
O-22*	Tiger Bay State Forest	FL	Volusia	29°10.22'N, 081°09.56'W	1			3	313
O-23*	Lake George State Forest	FL	Volusia	29°11.84'N, 081°30.55'W			1	1	364
O-24*	Deland	FL	Volusia	29°00.11'N, 081°13.25'W	1			1	365
*Callisia rosea* (Vent.) D.R. Hunt										
R-1	Along Chert Quarry Rd.	SC	Allendale	33°02.28'N, 081°28.26'W	1			3	245
R-2*	Heggie’s Rock Preserve	GA	Colombia	33°32.34'N, 082°15.09'W	1			3	321
R-3*	Lake Russel State Park	GA	Elbert	34°09.60'N, 082°44.42'W	1			3	237
R-4*	Bobbie Brown State Park	GA	Elbert	33°58.35'N, 082°34.64'W	1			3	238
R-5*	Elijah Clarke State Park	GA	Lincoln	33°51.22'N, 082°24.02'W	1			3	323
R-6	Fort Gordon	GA	Richmond	33°23.49'N, 082°14.54'W	1			3	240
R-7	Fort Stewart	GA	Tattnall	32°02.54'N, 081°48.84'W	1			4	244

### Collecting of specimens

The georeferenced data were used to relocate populations within the southeastern U.S.A.; additional localities were discovered by exploring similar habitats in protected areas and on private land. Collections on private land were made with permission of the land owners. Based on the georeferenced data, permits were obtained to collect in state parks, state forests, national parks, and protected areas of The Nature Conservancy and the U.S. Fish and Wildlife Service in Florida, Georgia, South Carolina, North Carolina, and Virginia (Table 1).

Mature individuals were sampled in the summers of 2012, 2013, 2014, and 2015. Only known localities with collection years between 1970 and 2012 were visited, unless the locality was in a protected area. This approach was used to increase the chances of finding intact populations but meant that we were unable to resample all of Giles’s (1942, 1943) locations. Voucher specimens were deposited at the University of Florida Herbarium (FLAS); collection numbers are provided in Table 1.

Population localities were surveyed for individuals with different growth habit and habitat; we then collected across that diversity. Contact zones between species, based on the georeferenced localities, were more intensively surveyed by searching for distinct morphological variation (habit, leaf, and flower) to increase the probability of encountering mixed cytotypes. Two to six live plants were collected per locality. Plants were removed with 15 cm of soil circumference to increase the survival rate and placed in plastic bags. At the Department of Biology, University of Florida greenhouse, plants were then potted in a soil mixture of 1:1 sand and potting soil (Pro-Mix) and were kept under natural light. During the period from December–March, the individuals of putative diploid *C. graminea* and *C. rosea* were given a four-month dormancy treatment at 4°C to mimic their natural habitat.

### Chromosome counts

Two individuals per cytotype of *C. graminea* were used as a control for flow cytometry analysis by counting chromosome numbers using established methods (see below). Previous studies of members of Commelinaceae found that cell division in root tips occurs at high frequency during late morning to early afternoon (Faden and Suda 1980). After a series of hourly collections, 2:00 pm was determined to be the optimal time for collecting root tips of *C. graminea*, *C. ornata*, and *C. rosea*.

Root tips were placed in 2 mM 8-hydroxyquinoline following Soltis (1980) for 24 hours at 4°C and then fixed in a 3:1 absolute ethanol-glacial acetic acid solution for 24 hours. Root tips were then placed in 70% ethanol and stored until needed at 4°C. Digestion of the root tips and spreading of the chromosomes on slides were performed following the methods of Kato et al. (2011). Chromosomes were stained with DAPI and visualized using a Zeiss Axio Imager M2 microscope (Carl Zeiss Microscopy LLC, Thornwood, NY, U.S.A.).

### Flow cytometry

Preparation of all samples for flow cytometry followed Roberts et al. (2009), in which each sample consisted of approximately 1 cm^2^ of fresh leaf tissue of *Callisia*; 0.5 cm^2^ dried leaf tissue of *Vicia faba* (26.9 pg) was used as an internal standard (Dolezel et al. 2007). Samples were finely chopped with a sharp single-edged razor blade in a petri dish for 2 min in 1 ml of cold lysis buffer (0.1 M citric acid, 0.5% v/v Triton X−100, 1% w/v PVP−40 in distilled water) (Hanson et al. 2005, Mavrodiev et al. 2015). After 20–30 sec of incubation on a cold brick that served as a cold chopping surface, each sample was further treated and measured based on the methods of Mavrodiev et al. (2015) on an Accuri C6 flow cytometer (BD Biosciences, San Jose, CA, U.S.A). In all, the ploidy of 300 samples was assessed in batches of 28 samples.

For the estimation of genome size, three plants of the same accession were analyzed using the Flow Cytometry Kaluza Analysis Software 1.3 (Beckman Coulter Life Sciences 2016). The relative DNA content was calculated using the ratio of the mean fluorescent peak of the sample to the mean fluorescent peak of the internal standard, multiplied by the genome size of the standard, *Vicia faba* (Dolezel et al. 2007).

## Results

### Georeferencing and collecting

**Figure 1.**
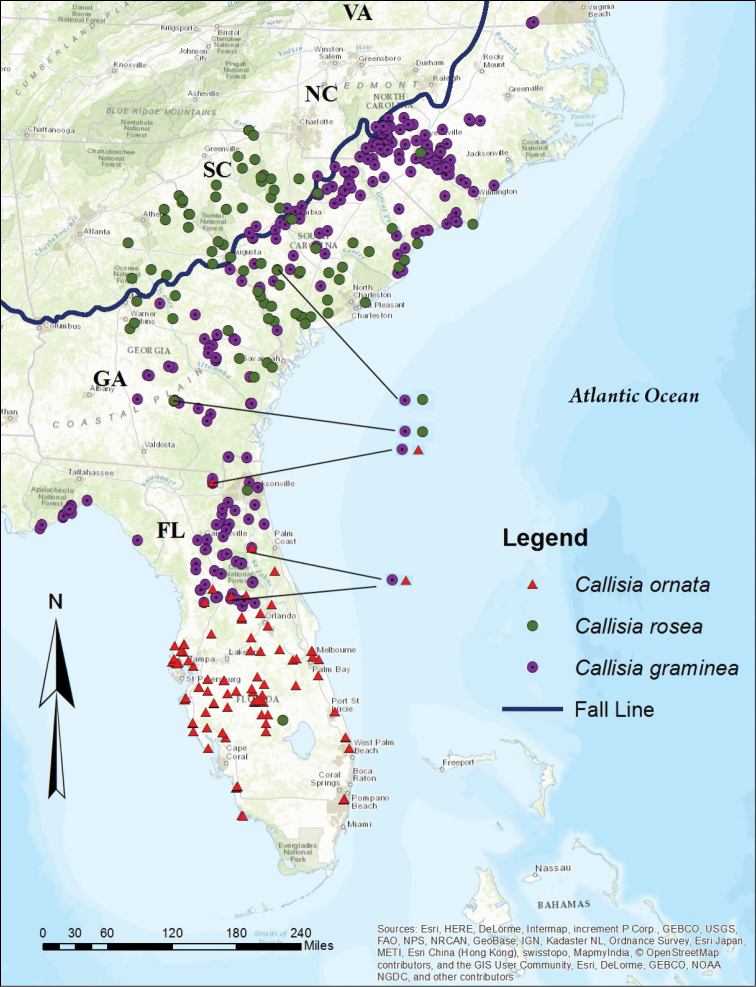
Distribution map of Callisia section Cuthbertia. Distribution of *Callisia graminea*, *C. ornata*, and *C. rosea* based on georeferenced data. Multiple species occurring in sympatry are designated by superimposed symbols; these locations are further indicated by black lines that highlight the symbols.

**Figure 2.**
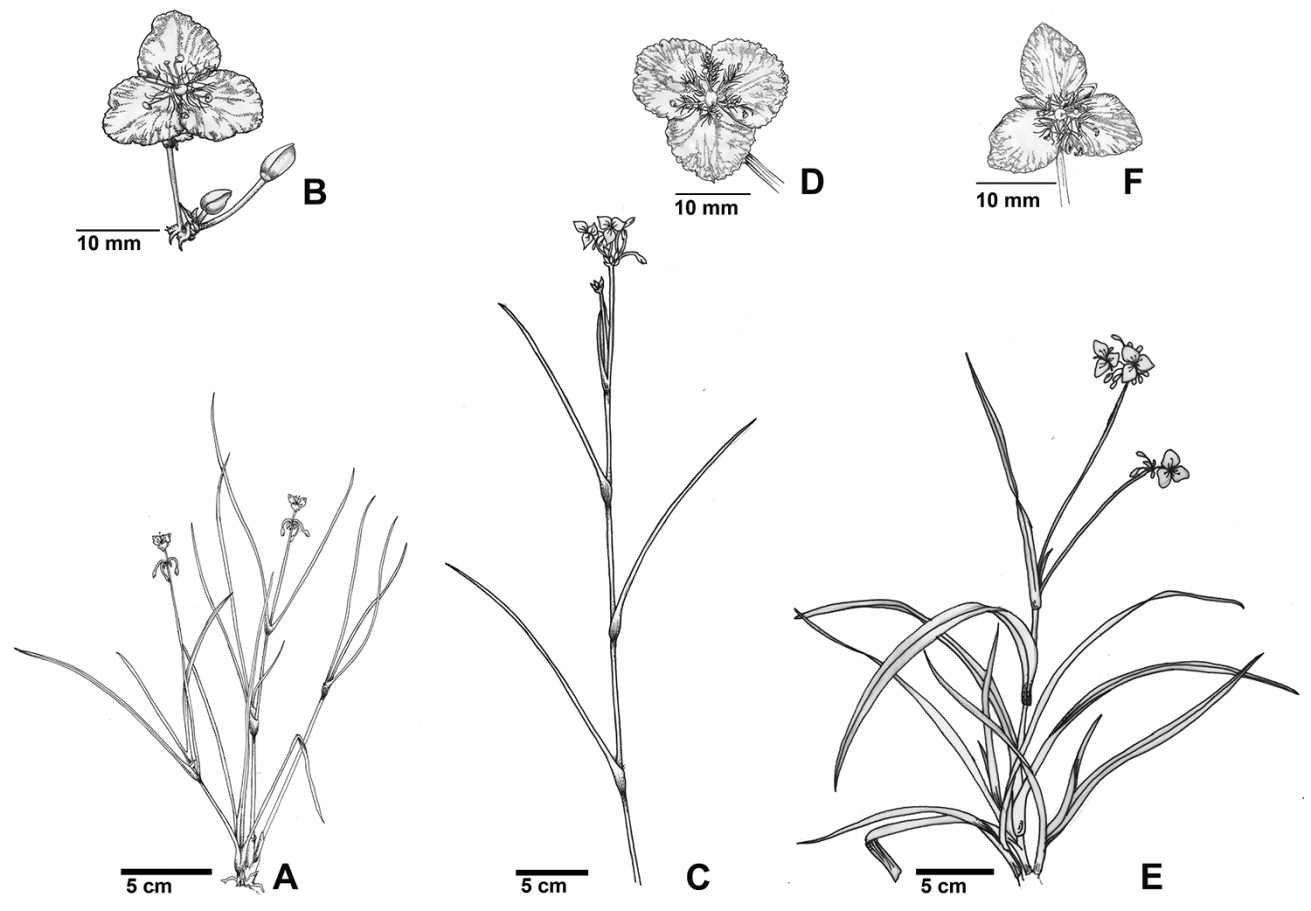
Habit of Callisia section Cuthbertia. **A** diploid *Callisia graminea* **B** diploid *C. graminea* flower **C** diploid *C. ornata* **D** diploid *C. ornata* flower **E** diploid *C. rosea* and **F** diploid *C. rosea* flower. Illustrations by Sofia Chang.

All GPS points obtained here were incorporated into a map with ARCGIS 10.4 (ESRI 2016) (Figure 1). The results show that *Callisia graminea* ranges from North Carolina to central Florida with an isolated population in southern Virginia. *Callisia rosea* occurs predominantly in South Carolina and Georgia, and *C. ornata* is found in central to southern Florida. Specimens were collected at 133 localities, of which 61 were known from the 436 georeferenced localities and 72 were newly discovered populations. A list of these localities is provided in Table 1, indicating the geographic origin, ploidal level with corresponding number of plants, total number of analyzed individuals, and voucher information for each sample. Illustrations of the habits of diploid *C. graminea*, *C. ornata*, and *C. rosea* are provided in Figure 2.

### Chromosome counts

**Figure 3.**
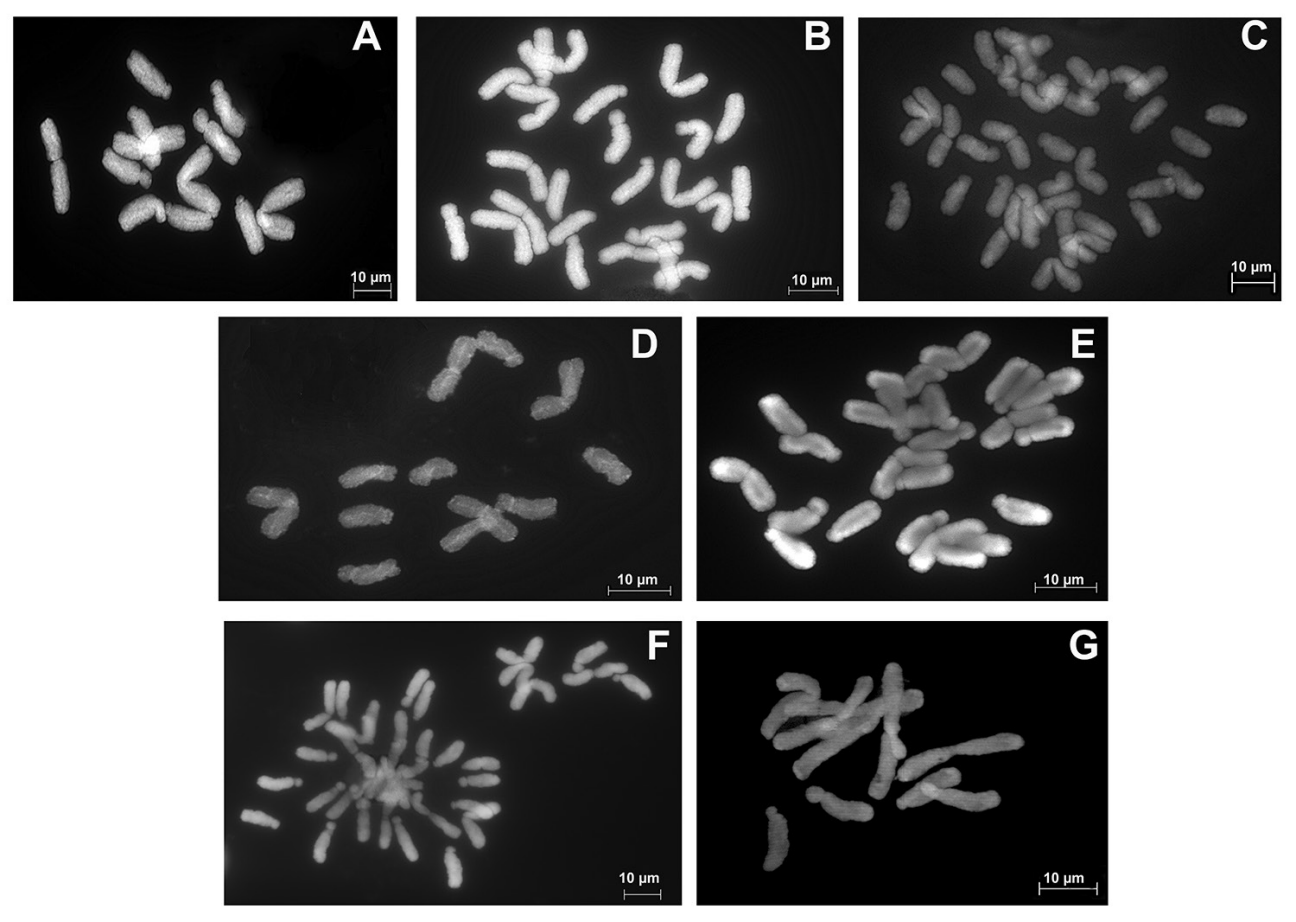
Mitotic metaphase chromosome spreads from root tips. **A** diploid *Callisia graminea* (2*n* = 2*x* = 12) **B** tetraploid *C. graminea* (2*n* = 4*x* = 24) **C** hexaploid *C. graminea* (2*n* = 6*x* = 36) **D** diploid *C. ornata* (2*n* = 2*x* = 12) **E** tetraploid *C. ornata* (2*n* = 4*x* = 24) **F** hexaploid *C. ornata* (2*n* = 6*x* = 36) and **G** diploid *C. rosea* (2*n* = 2*x* = 12).

Chromosome numbers were obtained for three individuals per cytotype in *C. graminea*, confirming the presence of 2*n* = 2*x* = 12 (diploids; Figure 3a), 2*n* = 4*x* = 24 (tetraploids; Figure 3b), and 2*n* = 6*x* = 36 (hexaploids; Figure 3c). The diploid and tetraploid counts were obtained for plants from known locations for which previous counts were available (Giles 1942, Kelly 1991). The hexaploids were discovered while counting spreads of putatively tetraploid *C. graminea* from Lake County, FL (Table 1). These 2*x*, 4*x*, and 6*x* individuals of *C. graminea* were then used as references in subsequent analyses using flow cytometry.

### Flow cytometry

**Figure 4.**
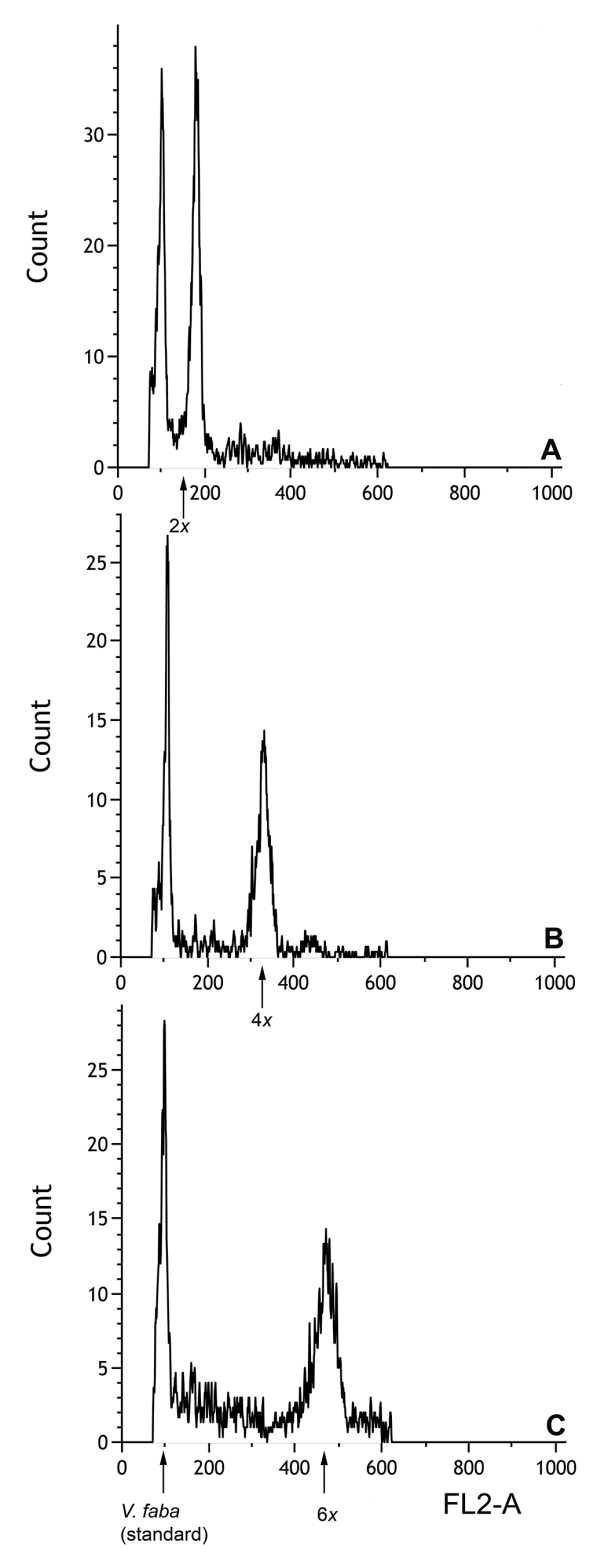
Histograms of fluorescence intensity (FL2-A) of propidium iodide-stained nuclei. **A** diploid *C. graminea* **B** tetraploid *C. graminea* and **C** hexaploid *C. graminea*. *Vicia faba* was used as the internal standard.

Ploidy was estimated via flow cytometry for 300 plants of *C. graminea* (representing 96 populations), *C. ornata* (from 23 populations), and *C. rosea* (from 7 populations). The results and the number of individuals analyzed per population are given in Table 1. Three distinct groups of fluorescence intensities were obtained from these analyses that were congruent with chromosome counts of diploid, tetraploid, and hexaploid *C. graminea*. Histograms for the cytotypes of *C. graminea* are shown in Figure 4. Results for 26 individuals (17%) of tetraploid *C. graminea* had a lower fluorescence intensity (suggesting a smaller genome size) than the remaining 83% of tetraploid *C. graminea*. The ploidy of the former plants was verified by chromosome counts, and all were tetraploid.

The relative genome size of individuals of *C. rosea* was similar to that of diploid *C. graminea* (2*n* = 2*x* = 12) (see below), confirming that our samples of *C. rosea* are diploid, in agreement with the literature (Giles 1942). Most individuals of *C. ornata* (2*n* =2*x* =12) were also inferred to be diploid, as expected based on previous counts (Giles unpublished), but our analysis also revealed previously unknown tetraploid (2*n* = 4*x* = 24) and hexaploid populations (2*n* = 4*x* = 36) of *C. ornata*. The latter were found in Seminole State Forest, FL, where they occur in sympatry with tetraploid individuals of *C. graminea*. All polyploid levels were verified with chromosome counts; chromosome spreads are depicted in Figure 3.

Genome size (2C-value) of cytotypes in Callisia section Cuthbertia was estimated; data are presented in Table 2 along with previously calculated genome sizes by Hertweck (2011) and Jones and Kenton (1984).

**Table 2.** Genome sizes (2C) of Callisia section Cuthbertia and their cytotypes and previously reported 2C-values. Voucher numbers apply only to the current study.

Species	Chromosomes	2C value (pg)	Hertweck 2011	Jones and Kenton 1984
*C. graminea* 2*x* (IEM 342)	2*n* = 12	41.75 ± 0.67		
*C. graminea* 4*x* (IEM 251)	2*n* = 24	78.55 ± 0.42		
*C. graminea* 6*x* (IEM 236)	2*n* = 36	122.86 ± 0.8		
*C. ornata* 2*x* (IEM 353)	2*n* = 12	48.51 ± 1.09		
*C. ornata* 4*x* (IEM 352)	2*n* = 24	87.99 ± 0.4		
*C. ornata* 6*x* (IEM 349)	2*n* = 36	129.73 ± 0.56		
*C. rosea* 2*x* (IEM 237)	2*n* = 12	43.70 ± 1.78	43.52	77.3

**Figure 5.**
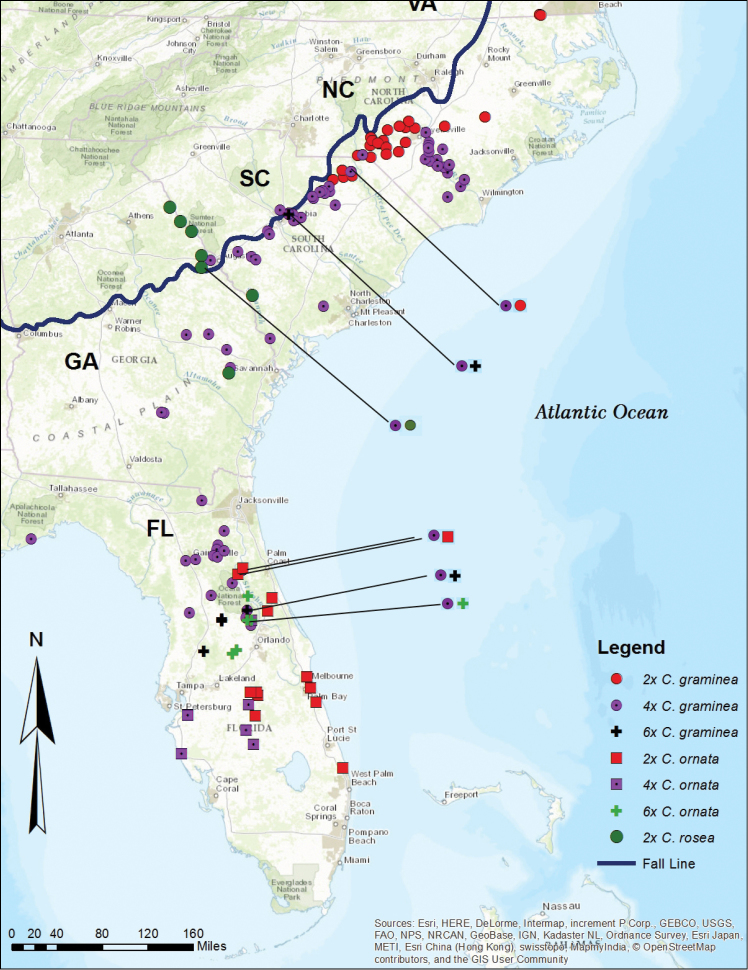
Distribution of cytotypic variation in *C. allisia* section Cuthbertia. Diploid *C. graminea* (red circles) ranges from Virginia to North and South Carolina; tetraploid *C. graminea* (purple circles) occurs along the coastal plain from North Carolina to central Florida; hexaploid *C. graminea* (black plus signs) is restricted to central Florida. Diploid *C. ornata* (red squares) occurs in eastern and central Florida; tetraploid *C. ornata* (purple squares) is restricted to central and western peninsular Florida; hexaploid *C. ornata* (green plus signs) is restricted to central Florida. *Callisia rosea* (all diploid; green diamonds) occurs along the Georgia – South Carolina border. Localities with multiple cytotypes or taxa are indicated by black lines. Note: The black plus signs are the hexaploids of *C. graminea*, and the green plus signs are hexaploids of *C. ornata*

*Distribution map* – Based on the flow cytometry data, the distribution of cytotypic variation among the 126 populations sampled [*C. graminea* (96 populations), *C. ornata* (23 populations), and *C. rosea* (7 populations)] was mapped (Figure 5). This map shows that diploid *C. graminea* is restricted to two disjunct areas: one in Franklin County, VA, and the second stretching along the Fall Line from North Carolina to South Carolina. Tetraploid *C. graminea* has a broader distribution that runs along the coastal plain from North Carolina to central Florida. Hexaploid *C. graminea* occurs in Lake and Hernando Counties, FL, and one individual was found in Richland County, SC. In South Carolina, one hexaploid *C. graminea* individual was found growing sympatrically with multiple tetraploid *C. graminea* plants. Based on extensive collecting, our observations suggest that the tetraploid *C. graminea* samples from North Carolina are the largest of this species, with clumps that exhibit a diameter of over 25 cm compared to plants in South Carolina, Georgia, and Florida, with a maximum diameter of 15 cm.

Diploid *C. ornata* occurs in eastern Florida (from Putnam through Martin Counties), and tetraploid *C. ornata* occurs in western Florida (Polk, Hillsborough, Highlands, and Lake Counties). Hexaploid *C. ornata* occurs in Lake and Volusia Counties in central Florida.

Diploid *C. rosea* occurs in the piedmont of Georgia and South Carolina with some scattered populations in the coastal plain.

## Discussion

*Georeferencing* – Callisia section Cuthbertia consists of three species native to the southeastern U.S.A., with three ploidal levels within *C. graminea* and *C. ornata* and diploids in *C. rosea*. The map of the geographic distribution (Figure 1) of all georeferenced voucher specimens depicts all specimens of *C. graminea*, *C. ornata*, and *C. rosea* without ploidal levels, collected from 1894 until present. *Callisia graminea* is the most widely distributed of all species in the genus, ranging from Virginia to Florida. *Callisia ornata* is restricted to Florida; although one specimen was recorded from Charleston County, GA, *C. ornata* was not found in Georgia in this study. *Callisia rosea* occurs mainly in Georgia and the Carolinas, but two herbarium specimens were found from Duval County and Highlands County, FL. The localities of these two herbarium specimens of *C. rosea* were vague, and *C. rosea* was not observed in Florida in this study.

*Flow cytometry and genome size* – Flow cytometry analysis of ploidal levels in 300 individuals from 126 populations together with 60 additional chromosome counts confirmed the presence of diploid, tetraploid, and hexaploid cytotypes of *C. graminea* and *C. ornata*. Significantly, tetraploid and hexaploid *C. ornata* were previously unknown. Our analysis also confirmed that *C. rosea* is diploid. However, Anderson and Sax (1936) and Ichikawa and Sparrow (1967) reported only tetraploids in *C. rosea*. This might be a misidentification of broad-leaved tetraploid *C. graminea* as *C. rosea*, as suggested by Giles (1942), who only detected diploids in *C. rosea*. Overall, three distinct fluorescent intensity peaks were seen in the histograms among the *C. graminea* and *C. ornata* cytotypes, with peaks for the tetraploids that are approximately twice the size of those of the diploids and for the hexaploids that are approximately three times those of the diploids. This general pattern of genome size increase in polyploids is to be expected relative to their diploid progenitors (Leitch and Bennett 2004).

It is interesting to note that 26 individuals (17%) of tetraploid *C. graminea* had a lower fluorescence intensity than the remaining 83%, suggesting a smaller genome size. The individuals with the smaller peak than that typical of other tetraploids were measured twice with the flow cytometer, and the results were consistent. The chromosome numbers of these samples were verified by chromosome counts, and all were tetraploid (2*n* = 4*x* = 24). Reductions in genome size in polyploids are common (Leitch and Bennett 2004), and in this study two hypotheses are possible: genome downsizing or the occurrence of multiple origins from parents having different genome sizes. Because this variation in genome size occurs among individuals within populations and because the individuals are not clustered in a single geographic area, we suggest that this variation in DNA content might be a result of genome downsizing, but this hypothesis requires further testing.

Genome size can be used, with other methods, to hypothesize putative progenitors of polyploids (e.g. Eilam et al. 2010). In diploid *C. graminea* the estimated 2C-value is 41.75 pg; the value for tetraploid *C. graminea* is 78.55 pg. According to Giles (1942), multivalent chromosome pairing was observed in tetraploid *C. graminea*, suggesting autopolyploidy. If tetraploid *C. graminea* is of autopolyploid origin, the expected DNA content would be 83.47 pg, but the observed DNA content of tetraploid *C. graminea* is 4.95 pg lower than the expected 2C-value. Newly formed polyploids usually possess a DNA content equal to the sum of the 2C-values of their progenitors (Bennett et al. 2000, Eilam et al. 2010). Over time, however, genome downsizing in polyploids relative to their progenitors is expected (Leitch and Bennett 2004), which seems to be the case in tetraploid relative to diploid *C. graminea*.

Due to the rarity of hexaploid *C. graminea* in South Carolina, we only calculated the 2C-value of hexaploids that occur in Florida. Hexaploid *C. graminea* may be of allo- or autopolyploid origin. If from allopolyploid origin, the expected 2C-value would be 127.06 pg, with diploid *C. ornata* (48.51 pg) and tetraploid *C. graminea* (78.55 pg) as the progenitors. The observed genome size of hexaploid *C. graminea* is 122.86 pg, which is lower than the expected value, again consistent with genome downsizing. In the case of an autopolyploid origin with tetraploid *C. graminea* (78.55 pg) as parent, we would expect a genome size of 117.83 pg, which is approximately 5 pg less than the observed 2C-value. Genome size data do not conclusively elucidate the origins of hexaploid *C. graminea*; both allo- and autopolyploidy are possible, and its origin requires further testing. However, Giles (1942) noted multivalent formation, generally indicative of autpolyploidy, in hexaploid *C. graminea*.

Tetraploid *C. ornata* has a 2C-value of 87.99 pg. It could be of autopolyploid origin with diploid *C. ornata* (48.51 pg) as the parent given that no other extant taxa are sympatric with it. However, the expected DNA content (97.02 pg) is at least 9 pg higher than observed; in contrast, when considering tetraploid *C. ornata* as a possible allopolyploid with tetraploid *C. graminea* (78.55 pg) and diploid *C. ornata* (48.51 pg) as parents (based on an unreduced gamete of the latter), the results (87.79 pg) are similar to the observed DNA content. These results therefore support allopolyploidy over autopolyploidy, yet further analyses are needed to clarify the origin of this cytotype.

Hexaploid *C. ornata* could be of allo- or autopolyploid origin. If allopolyploid, the expected genome size would be 127.06 pg with diploid *C. ornata* (48.51 pg) and tetraploid *C. graminea* (78.55 pg) as parents. The observed DNA content is 129.73 pg, which is slightly higher than the expected 2C-value. Alternatively, it could be an allohexaploid between tetraploid *C. ornata* (87.99 pg) and diploid *C. graminea* (41.75 pg), with an expected genome size of 129.74 pg, essentially identical to the observed value. In the case of autopolyploidy, we calculated an expected 2C-value of 145.53 if the value is 3 times that of diploid *C. ornata* (48.51 pg), 136.5 pg if tetraploid (87.99 pg) and diploid (48.51 pg) *C. ornata* are considered the parents, and 131.99 pg if a reduced and unreduced gamete of tetraploid *C. ornata* yield the hexaploid. The latter case is closest to the observed value, suggesting either that hexaploid *C. ornata* is of allopolyploid origin, or if an autopolyploid, it arose via the third possible mechanism outlined above; these hypotheses require further investigation.

Based on the Plant DNA C-values Database, http://data.kew.org/cvalues/ (Bennett and Leitch 2012), recorded species of Commelinaceae have a minimum 2C-value of 5.16 pg for *Commelina erecta* L.1753 and a maximum of 86.7 pg for *Tradescantia virginiana* L. 1753. The DNA content of hexaploid *C. graminea* and hexaploid *C. ornata* are currently the highest within Commelinaceae and Commelinales (Leitch et al. 2010) with 122.86 pg and 129.73 pg, respectively. Jones and Kenton (1984) reported that the 2C-value of *C. rosea* is 77.3 pg, with a chromosome count of 2*n* = 24, consistent with tetraploidy reported by Anderson and Sax (1936) and Ichikawa and Sparrow (1967); however, as noted above, Giles (1942) only detected diploids (2*n* = 12) for *C. rosea*, consistent with our results. The closest 2C-value to 77.3 pg is the 2C-value of tetraploid *C. graminea* with 78. 55 pg and 2*n* = 24 chromosomes; tetraploid *C. graminea* plants with broad leaves may be misidentified as *C. rosea* (Giles 1942). A voucher specimen of *C. rosea* from Jones and Kenton (1984) was not reported, so we cannot assess if the plant material used for the DNA content analysis was identified correctly. A misidentification is likely since the genome size estimation of Hertweck (2011) is close to our values. Likewise, previous tetraploid counts (Anderson and Sax 1936, Ichikawa and Sparrow 1967, Jones and Kenton 1984) may also be for tetraploid *C. graminea* plants that were misidentified as *C. rosea*. Alternatively, there may be cryptic tetraploidy in *C. rosea* that we failed to detect, but given our extensive sampling, we do not believe this to be the case.

*Distribution* – As shown in Figure 5, two isolated populations of diploid *C. graminea* were detected. One population is in Suffolk County, VA, and the other is in North and South Carolina. These two isolated populations may have been part of a once larger geographic range for diploid *C. graminea*, but due to heavy agricultural activities in this part of North Carolina, suitable habitats ranging from Johnston County to Northampton County were transformed to farmland (personal observation). This anthropogenic influence may have caused the separation of the two isolated groups of diploid *C. graminea*.

Tetraploid *C. graminea* ranges from the coastal plain of the Carolinas to central Florida, with additional populations in the Florida panhandle (Franklin County, FL). This cytotype is clearly more abundant than diploid *C. graminea*; it is usually found in xeric disturbed areas and exhibits a larger growth form than diploid *C. graminea*. These tetraploids were abundant in Bladen and southern Cumberland Counties, NC, which border the isolated locality of diploid *C. graminea* in North Carolina. These two areas (occupied by tetraploid and diploid plants, respectively) are separated by the city of Fayetteville, NC. Although diploid and tetraploid entities of *C. graminea* were reported to be geographically isolated (Bergamo 2003, Giles 1942, 1943, Kelly 1991), one tetraploid individual was found within a diploid population in Cheraw State Park, SC; this individual is morphologically similar to the surrounding diploid *C. graminea*. This finding supports Giles’s (1942) hypothesis that tetraploid *C. graminea* is an autotetraploid because it occurs consistently with diploid *C. graminea*. This hypothesis requires testing with molecular data.

The Fall Line runs essentially east-west through Georgia and from southwest to northeast in the Carolinas. Diploid *C. rosea* occurs on both sides of the Fall Line from Georgia to North Carolina. In Fort Gordon (Richmond County, GA), diploid *C. rosea* occurs in sympatry with tetraploid *C. graminea*. Although these two species occur in sympatry, hybrids were not observed at the site.

Diploid *C. ornata* is endemic to Florida, and tetraploid individuals of *C. ornata* occur in western Florida. These individuals may be autopolyploid, with diploid *C. ornata* as their progenitor. The distribution map in Figure 5 clearly supports the assumption of autopolyploidy, because there are no other *Callisia* species recorded in the region of diploid and tetraploid *C. ornata*. Morphologically, tetraploid *C. ornata* individuals show an increased axillary branching pattern, which is less common in diploid individuals. Axillary branching is a characteristic of *C. graminea*. Tetraploid *C. graminea* and diploid *C. ornata* are likely parents, through the union of one reduced gamete of tetraploid *C. graminea* and one unreduced gamete of diploid *C. ornata*.

In South Carolina, one hexaploid individual of *C. graminea* was found growing sympatrically with multiple tetraploid individuals of *C. graminea*. Hexaploid *C. graminea* in South Carolina appeared to be rare, and in 1942 only one individual was reported by Giles (1942). These rare hexaploid individuals may be allopolyploids, with diploid *C. rosea* and tetraploid *C. graminea* as their parents or autopolyploids with tetraploid *C. graminea* as their progenitor. Regarding allopolyploidy, *C. rosea* was not found sympatrically with tetraploid *C. graminea* in South Carolina; however, from the map of georeferenced specimens (Figure 1), there is a significant overlap of distribution between tetraploid *C. graminea* and diploid *C. rosea* in the Carolinas. With regard to autopolyploidy, individuals may have resulted through the union of one reduced and one unreduced gamete of tetraploid *C. graminea* given that no other *Callisia* species were observed in the population.

In Lake and Hernando Counties, FL, hexaploid individuals exhibited intermediate morphological characteristics between *C. graminea* and *C. ornata*. Some populations had typical tetraploid *C. graminea* or diploid *C. ornata* characteristics (Figure 2). Two forms were distinguished based on habit: (1) hexaploid *C. graminea* and (2) hexaploid *C. ornata*. Hexaploid *C. graminea* and one of its possible progenitors, tetraploid *C. graminea*, grow in sympatry at the Seminole State Forest, and hexaploid *C. ornata* was found growing with tetraploid *C. graminea* at the entrance to Brantley Branch Rd. (Seminole State Forest). The co-occurrence of hexaploids and tetraploids suggests that the hexaploids may be of allopolyploid origin. Hexaploid *C. graminea* was also collected at Lake Griffin State Park, Edward Rd., Lady Lake, and Seminole State Forest, FL. In Dunns Creek State Park and Welaka State Forest, diploid *C. ornata* and tetraploid *C. graminea* occur in sympatry; however, hexaploids were not found in these contact zones.

The rare hexaploid collected in South Carolina is most likely independently evolved from the hexaploids from Florida, and this entity from South Carolina could be either an allo- or autopolyploid. If allopolyploid, one likely parent, *C. rosea*, only occurs in Georgia and the Carolinas; if autopolyploid, the likely parent is tetraploid *C. graminea*. The hexaploid entities of Florida might be allopolyploid due to the intermediate morphological characters, with diploid *C. ornata* and tetraploid *C. graminea* as progenitors.

Callisia graminea forma leucantha, which was reported near Tampa, FL, was not found, but one white-flowered tetraploid individual of *C. graminea* was encountered among pink-flowered individuals in each of the following three locations: Sesquicentennial State Park, SC; Chesterfield Co., SC; and Tate’s Hell State Forest, FL. One white-flowered individual of diploid *C. rosea* was found in Heggie’s Rock Preserve, Appling, GA. White flowers reflect an absence of anthocyanins, which may result from mutations in any of the genes in the anthocyanin pathway or from lack of expression of potentially functional genes (Ho and Smith 2016, Rausher 2008). In Callisia section Cuthbertia, variation in flower color is common, but there is no association between color and ploidy within or among populations. Loss of anthocyanin pigments seems to occur sporadically within this complex.

Morphological and molecular analysis is an important next step in unraveling the complex relationships among cytotypes of Callisia section Cuthbertia. This work will allow us to reveal the parentage, evolutionary history, and the evolutionary role of all cytotypes within Callisia section Cuthbertia.

## Supplementary materials

Supplementary material 1 Georeferenced data points Data type: occurence This dataset is made available under the Open Database License (http://opendatacommons.org/licenses/odbl/1.0/). The Open Database License (ODbL) is a license agreement intended to allow users to freely share, modify, and use this Dataset while maintaining this same freedom for others, provided that the original source and author(s) are credited.Iwan E. Molgo, Douglas E. Soltis, Pamela S. Soltis
